# Folding, Stability and Shape of Proteins in Crowded Environments: Experimental and Computational Approaches

**DOI:** 10.3390/ijms10020572

**Published:** 2009-02-13

**Authors:** Antonios Samiotakis, Pernilla Wittung-Stafshede, Margaret S. Cheung

**Affiliations:** 1 Department of Physics, University of Houston, Houston, Texas 77204, USA; E-mail: asamiotakis2@uh.edu; 2 Department of Chemistry, Umeå University, Umeå 90187, Sweden

**Keywords:** Macromolecular crowding, Ficoll^®^ 70, energy landscape theory, off-lattice model, excluded volume effect, protein folding mechanism, spectroscopy

## Abstract

How the crowded environment inside cells affects folding, stability and structures of proteins is a vital question, since most proteins are made and function inside cells. Here we describe how crowded conditions can be created *in vitro* and *in silico* and how we have used this to probe effects on protein properties. We have found that folded forms of proteins become more compact in the presence of macromolecular crowding agents; if the protein is aspherical, the shape also changes (extent dictated by native-state stability and chemical conditions). It was also discovered that the shape of the macromolecular crowding agent modulates the folding mechanism of a protein; in addition, the extent of asphericity of the protein itself is an important factor in defining its folding speed.

## 1. Introduction

It is most often assumed that protein-folding processes observed in dilute buffer solutions *in vitro* also represent the *in vivo* scenario. However, the intracellular environment is highly crowded due to the presence of large amounts of soluble and insoluble biomolecules (including proteins, nucleic acids, ribosomes, and carbohydrates). The presence of many large molecules means that a significant fraction of the intracellular space is not available to other macromolecular species. It has been estimated that the concentration of macromolecules in the cytoplasm is in the range of 80 to 400 mg/mL [1,2]. All macromolecules in physiological fluids collectively occupy between 10 and 40% of the total aqua-based volume [3,4]. The term ‘macromolecular crowding’ implies the non-specific influence of steric repulsions of macromolecules on specific reactions that occur in highly volume-occupied media [5–7]. Due to excluded volume effects, any reaction that amplifies the available volume will be stimulated by macromolecular crowding [8–11], as partly shown in the thermodynamic analysis of protein equilibrium in multicomponent systems (see a review article by Eisenberg [12]). It is proposed that the major result of macromolecular crowding on individual proteins is an indirect stabilization of the folded state that is due to destabilization of the unfolded polypeptide because of compaction[10,13]. The influence of macromolecular crowding on thermodynamics and kinetics of biological processes in cellular media has been recognized since the 1960s through pioneering work of Ogston and Laurent [14,15]. In the last 10 years, experimental and theoretical work has demonstrated large effects of crowding on the thermodynamics and kinetics of many biological processes *in vitro*, including protein stability, binding, folding, and aggregation [1,13,6–20]. It is also realized that conventional equations for biochemical reactions based on the law of mass action may break down at *in vivo* conditions [21].

Whereas theoretical simulations on crowding have focused on small proteins or peptides [13] experimental crowding studies in solution have mostly involved large, complex proteins (*i.e.,* multi-domain and/or disulfide containing) and often extreme solvent conditions (such as acidic pH). A few studies have focused on the ability of crowding agents to induce conformational changes in unfolded states of proteins. For example, unfolded cytochrome c was found to adopt a molten globule state in the presence of crowding agents at low pH [22] and two intrinsically unstructured proteins (FlgM and a variant of RNase T1) were discovered to fold in crowded conditions [23,24]. Using a combination of *in vitro* and *in silico* approaches, we have focused on the effects of volume exclusion from surrounding molecules (i.e. macromolecular crowding) on the behaviors of some small, single-domain proteins that fold with simple mechanisms in dilute solutions.

In this paper we summarize our recent efforts in this direction in which the size of macromolecules is much greater than solvent molecules. Our main concern focuses on the understanding of protein dynamics under cell-like conditions in which proteins are restricted in a tight space formed by large surrounding macromolecules (unlikely to compete for preferential solvation occupancy of a protein domain as small cosolutes do [20]). Our research on protein dynamics and conformations of proteins with varying structural geometries under macromolecular crowding suggested that the intrinsic shape of a protein may be important to its statistical properties in a jam-packed polydisperse environment, which will be included in Sections (2) to (5). These studies motivated us to further revisit the relationship of folding kinetics and its structural characteristic parameter in a form of contact orders, which will be included in Section (6). Below follow chapters on (2) how crowded conditions are created *in vitro* and *in silico* and the model proteins used, (3) the effect of crowding on equilibrium properties of a spherical protein, (4) how the shape of the crowding agent can influence folding trajectories, (5) the dramatic effect crowding has on the shape of a prolate protein, (6) the role of asphericity in defining folding speed, and finally (7), conclusions with a discussion including future directions.

## 2. Crowded conditions *in vitro* and *in silico* and protein models

**Figure 1.**
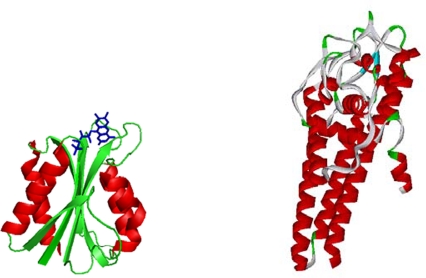
Cartoon structures of flavodoxin (left) and VlsE (right) indicating secondary structure motifs (using crystal structures). The cofactor, FMN, in flavodoxin is shown in stick (but removed in experiments presented here).

Crowded conditions can be created experimentally by adding inert synthetic or natural macromolecules, termed crowding agents, to the systems *in vitro*. Ficoll^®^ 70 (*i.e.,* a highly branched copolymer of sucrose and epichlorohydrin building blocks) and Dextran 70 (*i.e.,* a flexible long-chain poly(D-glucose) with sparse and short branches) are polysaccharides that are inert, polar and do not interact with proteins. Using a hard-particle approximation for crowders [9,10], Ficoll^®^ behaves like a semi-rigid sphere (radius ∼55Å) whereas Dextran is modeled as a cylindrical object [9,25,26]. Both polymers are attractive and they mimic macromolecules that may be present in the biological setting where proteins normally fold. The model proteins we have studied were strategically selected to include variation in secondary structure motifs and shapes (Figure 1): *Borrelia burgdorferi* VlsE (341 residues) has 50% α-helices and the rest is mostly unstructured loops [27,28]*Desulfovibrio desulfuricans* flavodoxin (148 residues), in contrast, has a mixed α/β topology with a flavin mononucleotide (FMN) cofactor (Figure 1) [29]. The FMN can be removed creating the apo-form, which has the same structure as the holo-protein. Importantly, both proteins have been characterized previously in our laboratory in terms of chemical and thermal unfolding behaviors in dilute solutions [27,30,31]; both proteins have rather low thermal (T_m_ of ∼50 °C; pH 7) and thermodynamic (ΔG_U_ of 15–20 kJ/mol; pH 7,20 °C) stabilities. Both proteins unfold in two-state-like equilibrium reactions in dilute solutions pH 7; thus, only native, unfolded and the high-energy transition states are involved.

To study macromolecular crowding effects, we use a statistical physics approach based on the Energy Landscape Theory [32,33] in combination with molecular simulations [34,35]. Low-resolution representations of a protein that keep its essential features in crystal structures are used for simulations. Crowding agents are modeled as hard spheres [36] and they provide non-specific (hard-core) interactions in molecular simulations [13]. Details about the protein models and the energy functions used for proteins and crowders are provided in the Appendix. Together with a method to reconstruct fine-grained protein models from low-resolution ones [37,38], the combination of *in vitro* and *in silico* studies of the same protein system allows us to make unique conclusions with near all-atomistic detail.

## 3. Effect of crowding on equilibrium properties of a spherical protein

**Figure 2.**
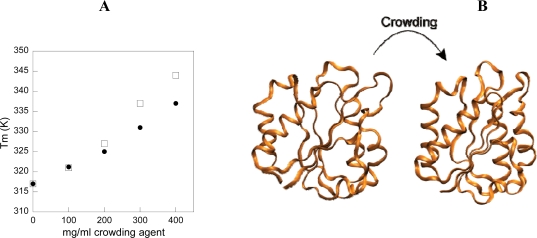
(A). Thermal midpoints for apo-flavodoxin unfolding (pH 7, Hepes buffer) as a function of amount of crowding agents Ficoll**^®^** 70 (spheres) and dextran 70 (circles). (B). Illustration of the structural changes that occur in folded apo-flavodoxin upon placing it in crowded conditions. The protein becomes more compact overall, the helices exhibit less terminal fraying and pack better towards the central sheet. High resolution structures are created from low resolution simulation conformations using the program SCAAL [37, 38].

To investigate the consequences of macromolecular crowding on the behavior of a globular protein, we performed a combined experimental and computational study on *D. desulfuricans* apo-flavodoxin [36]. Far-UV circular dichroism (CD) at 222 nm was used to probe thermal unfolding of apo-flavodoxin as a function of increasing Ficoll^®^ 70 (and dextran 70) concentrations up to 400 mg/mL, pH 7 (Figure 2A). All reactions appear as single, cooperative transitions and are reversible. The more Ficoll^®^ 70 present in the samples; the higher is the thermal midpoint (T_m_) for apo-flavodoxin. In fact, T_m_ increases from 45 to 65 °C, going from 0 to 400 mg/mL Ficoll^®^ 70 in Hepes buffer, pH 7. Surprisingly, we find that the negative far-UV CD signal of the folded protein (pH 7.0,20°C; condition where in essence 100% of all molecules are in the folded state) grows larger when more Ficoll^®^ 70 is added, suggesting gain of secondary structure in the folded ensemble of molecules as a function of added crowding agent. Secondary structure estimations based on the far-UV CD spectra of folded flavodoxin reveal that the helical content rises up to 20%, while the random coil contribution shrinks more than 10%, when going from 0 to 400 mg/mL Ficoll^®^ 70 conditions in buffer. In addition, we found that Dextran 70 also induces additional structure in folded apo-flavodoxin. However, the stabilizing osmolytes glycerol and sucrose (the latter is the building block of Ficoll^®^), i.e., small molecules, do not alter the structural content of the folded state, although their presence results in increased resistance to thermal perturbation. As judged by far-UV CD, the effects of Ficoll^®^ 70 are minor with respect to the structural content of the unfolded ensemble of polypeptides.

To compare with the experimental data, we computed thermodynamic properties and simulated the free-energy landscape for apo-flavodoxin at different temperatures, with and without hard-sphere Ficoll^®^ 70 particles at a volume occupancy of φ_c_=25% [36] (See Appendix for simulation and modeling details). The computational analysis from molecular simulations are in good agreement with the *in vitro* data: the simulations demonstrate that, in the presence of 25% volume occupancy of spheres, folded apo-flavodoxin is thermally stabilized and the free energy landscape shifts to favor more compact and structured species in both folded and denatured states. This type of folded-state change was not observed in a previous investigation of the WW-domain [13]. This difference may be due to the fact that flavodoxin is longer (148 residues) and contains more complex secondary and tertiary structures than the WW-domain (34 residues).

To reveal the molecular origin of the crowding-induced protein compaction and increased structural content, we derived difference contact maps of the folded states of apo-flavodoxin between the φ_c_=25% and the bulk conditions. Inspection of the map reveals that the compaction of folded apo-flavodoxin stems from improved interactions between the surrounding helices and the core β-sheet, as well as from less helix fraying in the terminal helices (Figure 2B). The extension of helices agrees well with the far-UV CD data that implied more α-helical content in folded apo-flavodoxin at crowded conditions.

In agreement with our findings, using an equilibrium statistical-thermodynamic model, Minton has predicted that macromolecular crowding should increase protein thermal stability (T_m_) by a magnitude of about 5 to 20 °C at physiological solute conditions [9,39]. In these papers, it is stated that the major effect of excluded volume in concentrated solutions of inert macromolecules is to stabilize the native states of proteins by preferentially destabilizing the unfolded states. By making the denatured state more compact, and thereby less energetically favorable, the native state is indirectly stabilized [8–10]. However, our experimental and computational observations of structural changes in both the folded and denatured states of apo-flavodoxin indicate that direct crowding effects on the folded protein molecules are feasible. We have observed both a compaction of the overall size of the native protein (*i.e.,* effects on the computational R_g_) and a more native-like structure (*i.e.,* more negative experimental far-UV CD signal and computational Q value closer to one) for apo-flavodoxin in the presence of crowder. Based on our findings [36,40], we propose that native-state structural effects caused by macromolecular crowding may be common *in vivo* for globular proteins that exhibit marginal stability.

## 4. Shape of crowding agent influences folding routes

To quantify macromolecular crowding effects on protein folding mechanisms, we also investigated the folding energy landscape of apoflavodoxin in the presence of inert macromolecular crowding agents using *in silico* and *in vitro* approaches [41]. By using coarse-grained molecular simulations [42] and a topology-based energy function that best represents protein folding through few intermediates [43] (see Appendix for simulation and modeling details), we investigated the effects of increased volume fraction of crowding agents (φ_c_) as a function of crowding agent geometry (sphere that models Ficoll^®^ 70 or sphero-cylinder that models Dextran 70).

We observed a change in the folding pathway by changing the geometry of the crowding agents. With our *in silico* model of apo-flavodoxin, we find that in the absence of crowding agents correct contact formation around the third β-strand in the central β-sheet is crucial in order to continue folding to the native state, in agreement with previous experimental findings [44]. Upon the addition of spherical crowding agents (corresponding to Ficoll^®^ 70), we observe an off-pathway folding route that favors early formation of the first terminal β-strand that dominates at high ϕ_c_. This causes topological frustration in protein models [45,46] (i.e., Topological frustration is a phenomenon in which during the evolution of folding, misfolded structures - despite the formation of native contact pairs - cannot directly reach the folded state. The formation of these native contacts hinders a sequential folding process and in order to reach the native state, these structures have to first unfold and then refold correctly) and the protein must unfold in order to allow the third β-strand to recruit long-range contacts to complete the central β-sheet. Surprisingly, when the spherical crowding agents are replaced by dumbbell-shaped ones, the topological frustration in apo-flavodoxin’s folding routes vanishes. In agreement with different mechanisms, stopped-flow mixing experiments with purified *D. desulfuricans* apoflavodoxin *in vitro* show that folding in buffer and in Ficoll^®^ 70 involves rapid formation of an intermediate with ∼30% folded-like secondary structure, that is followed by a slow final folding phase; in contrast, in Dextran 70 apo-flavodoxin’s burst-phase intermediate now includes ∼70% folded-like secondary structure.

**Figure 3.**
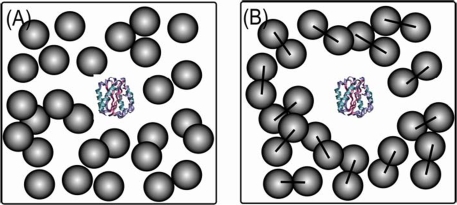
Schematic illustration of the available space to a protein in the presence of crowding agents in different geometries. Given the same volume fraction of crowding agents, the space available to a protein is greater in (B) than in (A). A dumbbell crowder in (B) is made out of two spherical crowders in (A).

This leads us to conclude that folding routes are sensitive to the space available [47] to the protein in the presence of crowding agents as illustrated in Figure 3. Despite that the total volume of crowders remains the same (ϕ_c_,), the space available to a protein (1- ϕ_c_)/ρ, where ρ is the number density of crowders, can differ. (1- ϕ_c_)/ρ in Figure 3B is two-fold higher than that in Figure 3A because the number density is reduced by half when two Ficoll^®^ spheres are brought together into one dumbbell-shaped crowder. The role of an available space to a protein will be most important at high ϕ_c_. This is because the density fluctuations of crowding agents [48] of varying shapes can alter the size of an average void that accommodates a protein. As the shapes and sizes of the structural ensemble of polypeptides change during the evolution of protein folding, the surrounding crowders of different geometries may have strong effects on a protein’s folding mechanism.

This idea explains the presence of topologically-frustrated protein structures at high ϕ_c_. The average void formed by the density fluctuation of the (spherical) crowding agents is quite small; therefore, rodlike elongated unfolded ensemble structures with smaller cross sections are being populated. The elongated unfolded ensemble structures that cause topological frustration in the folding process can be diagnosed by the folding route analysis [49,50]. However, upon changing the geometry of the crowding agent from spherical (Figure 3A) to dumbbell (Figure 3B), the elastic deformations of the unfolded polypeptides due to the crowding agents are relieved and this impacts partitioning between possible folding pathways. Thus, upon manipulation of crowded conditions, it appears that folding routes experiencing topological frustrations can be either enhanced or relieved [41]. We propose that the shape of surrounding macromolecules may influence protein folding kinetics in living cells as well.

## 5. Dramatic effect of crowding on shape of a prolate protein

How the crowded environment inside cells affects proteins with *aspherical shapes* is a vital question since many proteins and protein-protein complexes *in vivo* adopt anisotropic shapes. We addressed this by combining computational and experimental studies of the football-shaped protein *B. burgdorferi* VlsE in crowded, cell-like conditions [38]. The *B. burgdorferi* spirochete is the causative bacteria of Lyme disease; VlsE is proposed to be an important virulence factor upon mammalian infection and a specific diagnostic test for Lyme disease was derived from a 26-residue peptide region in VlsE named IR_6_ [51].

Spectroscopic methods were used to monitor urea-induced unfolding of VlsE in the presence of Ficoll^®^ 70 up to 100 mg/mL (pH 7, 20 °C). There is a shift in the transition midpoint to higher urea concentrations, and the unfolding-free energy increases in the presence of Ficoll^®^ 70. The mechanistic origin of the effects on stability was revealed via dynamic folding/unfolding experiments as a function of urea. VlsE folds in a two-state kinetic reaction in urea, both with [38] and without [27] crowding agents. In the presence of 100 mg/mL Ficoll^®^ 70, the folding speed in water is 3-fold faster than without Ficoll^®^ 70 whereas there is no effect on the unfolding speed. The *in vitro* VlsE kinetics are in excellent agreement with theoretical predictions based on the small WW domain peptide [13] where the folding kinetics for the WW domain was predicted to increase up to three-fold after the inclusion of non-interacting spheres as crowding agents. Our *in vitro* data on the much larger VlsE (341 residues versus 34), is the first experimental validation of this prediction.

Like in the case of apo-flavodoxin, there is an increase of VlsE helical structure in the presence of increasing levels of Ficoll^®^ 70. In fact, the helical content in VlsE increases from 50% (as in crystal) to 80% upon inclusion of 400 mg/mL Ficoll^®^ 70 [40]. In contrast to in buffer, at high levels of Ficoll^®^ 70 in the presence of urea, a non-native VlsE species is populated. The far-UV CD spectrum of this species has a negative peak around 220 nm which indicates various β-structures [52]. The same species is detected in thermal experiments with VlsE in the presence of 200 mg/mL, or more, Ficoll^®^ 70. Moreover, when refolding of VlsE denatured in buffer with 2 M urea (no Ficoll^®^ 70) was triggered by dilutions into buffer solutions containing at least 250 mg/mL Ficoll^®^ 70, the non-native β-rich species was again detected.

**Figure 4.**
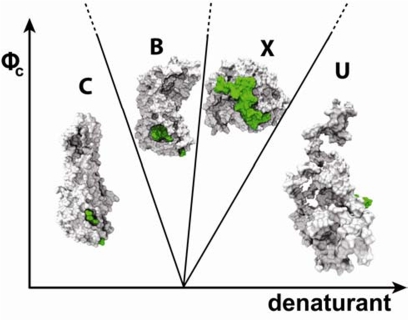
Phase diagram of VlsE conformations as a function of the volume fraction of crowding agents, ϕ_c_, and ambient perturbation (thermal or chemical denaturation). The structures shown are surface plots of high-resolution structures created from low-resolution configurations. The antigenic region, IR6, is shown in green.

To explain the physics behind these *in vitro* observed structural changes, we used computational simulations to study the energy landscape of VlsE at varying crowding conditions and temperatures [38] (See Appendix for simulation and modeling details). VlsE protein is represented by a coarse-grained model. An energy function that captures multiple intermediates is used for the investigation of molecular simulations. Characterization of its statistical properties under macromolecular crowding is carried out by plotting the folding energy landscape as a function of the overlap function (χ) and the radius of gyration (R_g_). Overlap function (χ) is a microscopic measure of the similarity to the crystal structure [28] where χ=0 for being similar and χ=1, otherwise. Several distinct populated states in the energy landscape of VlsE can be distinguished by the shape and asphericity parameters. At low temperature in bulk, the shape of the dominant VlsE ensemble conformations resembles a football (labeled as “C” in Figure 4). Second to the C state are ensemble conformations “B” that are similar to a bent bean. The ensemble conformations of the least populated state, named X, are spherical and most divergent from the crystal structure. 2D free energy diagrams for VlsE as a function of χ and R_g_ at different temperatures reveal that at low T, ϕ_c_ stabilizes the C state. At a somewhat elevated T, the population of the bean-like B conformations starts to dominate over the C state. To reveal where in VlsE changes occur in the bean (B) structure, we computed the number of non-native excess helical contacts, H_nn_, using difference contact maps derived from the coarse-grained ensemble structures. H_nn_ for the bean structure at ϕ_c_=15% and k_B_T/ε=1.13 is 32 which corresponds to an increase of helical structure of ∼30%. The new helical interactions in B are found in loop regions and in the end of helices. This finding of an increase in helical content due to crowding agreed with the experimental CD measurement on VlsE in the presence of high content of Ficoll^®^ 70.

When T is further increased and high ϕ_c_ is reached (ϕ_c_=25%), the spherical X state becomes most populated. This species is formed by “breaking” the bean-like structure in the middle; thereby both of the pointy ends of the protein are brought inwards, resulting in a compact spherical structure. Contact maps reveal that X has lost native helical interactions. Instead, it attained more nonnative interactions that appear to correspond to contacts for β-strands. This observation agrees with experimental CD measurement in which the helical content diminishes in the presence of both Ficoll^®^ and urea. The competition of the stabilizing crowding effects and the destabilizing factor of thermal/urea denaturation on the folding dynamics of VlsE produces a rich ϕ_c_-T/urea phase diagram in Figure 4, summarizing the agreement of both computational and experimental finding. This is why we speculate that the crowded environment of VlsE protein in the crystal lattice is far from the crowded environment inside a polydisperse cell, because crystallization does not result in the same protein structure as found here in crowded solution conditions in both experiments and simulations.

When VlsE is attached to the intact *B. burgdorferi* spirochete, the dominant antigenic region IR_6_ is cryptic (13% exposure to solvent in crystal structure). Remarkably we find that this stretch is flanked out of the variable regions upon shape changes and becomes surface-exposed in the X state of VlsE. Using the reconstructed high-resolution all-atomistic model of X, we computed the accessible solvent area of the IR_6_ region to be 31.7% ± 1.3% (Figure 4). Upon the release of VlsE into the host, the crowded milieu may trigger shape changes (mixture of B and X conformations) allowing for IR_6_ exposure: this explains why lymphocyte receptors can access this region *in vivo* and trigger antibody generation in Lyme disease patients [51,53].

## 6. Role of shape in defining protein-folding speed

Our work on folding of spherical and aspherical proteins under high level of crowding conditions inspired us to investigate further how the shape of a protein affects its folding dynamics and motion in a cell. To begin to address this issue, first we asked if folding kinetics of proteins that have shapes far from the resemblance of a sphere would be different from folding of spherical proteins. Therefore, we revisited the famous relationship between topology of a protein and folding rate, discovered by Plaxco *et al.* in 1998 [54]. If shape indeed matters to a protein’s folding behavior, we may detect this influence by examining structural characteristics of proteins that correspond to outlier points in the fitted linear relationship between the contact order of the native-state of a protein and its folding kinetics.

**Figure 5.**
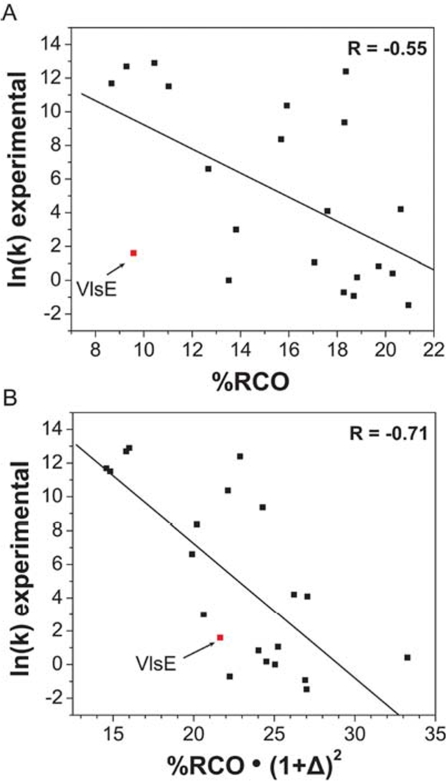
Linear relationship between experimental folding rates (ln *k*) vs. relative contact order (RCO) in (A) and ln *k* vs. RCO(1+Δ)^2^ in (B). Protein structures that are poorly correlated from Liang’s study are chosen [55]. The position of data for VlsE is marked red in both graphs to illustrate a stronger correlation once asphericity (Δ) is considered.

Plaxco *et al.* demonstrated a significant correlation between the average sequence separation between contacting residues in the folded state (RCO or relative contact order) and the natural logarithm of the folding rate (ln *k*) for a large set of unrelated single-domain proteins [54]. However, this correlation is not strong as data points for many proteins lie far apart from the straight line. VlsE is an excellent example since its folding speed cannot be predicted correctly by either RCO or other models [27,28] (Figure 5A). Other groups have subsequently proposed more complicated definitions of protein contact orders in attempts to show greater correlations to folding rates. Liang *et al.* [55] used a sophisticated geometry contact order parameter based on exhaustively enumerating spatial contacts that aimed to describe more complex protein structures, particularly those that account for large deviations from the correlation. By doing this, they achieved a higher correlation of two-state and multi-state folding proteins which implied that the geometry of a protein may be important for its folding kinetics.

We introduce a geometry factor in the contact order calculation by taking the asphericity (Δ; Δ=0 a sphere, Δ=1 a rod) into account without exhaustive computation of spatial contacts. We chose the protein data set in Liang’s study that was evidently deviated from the linear correlation established by Plaxco *et al.* (21 out of 45 protein structures). If the experimental folding rates (ln *k*) deviate the fitted linear relationship by more than 2.5 folds, the protein is selected for our study. Interestingly the majority of these proteins (∼70%) have Δ > 0.1 indicating that their shape is aspherical. The correlation coefficient of ln *k* vs. original RCO of this set alone is R=–0.49 (Figure 5A). When we modify the linear relationship of ln *k* and RCO by multiplying with a higher order term (1+ Δ)^2^ to RCO, we find that these outlier data points become better correlated (R=–0.68) in a ln*k* vs. RCO(1+ Δ)^2^ plot (Figure 5B). We also tested that the multiplication of (1+Δ)^2^ has no effect on the rest of protein sets that correlate well with RCO. It is because the majority of these well-behaved protein sets are mostly spherical (Δ∼0) and the asphericity is simply a higher-order correction.

This analysis with a simple function of Δ suggests that protein shapes may play a key role in defining the folding rates. This finding will be particularly important to protein folding *in vivo* because in a polydisperse crowded environment, statistical properties and dynamics of a protein will be influenced by its ambient macromolecules (see section 4). Despite similar sizes, proteins with distinctly different shapes could have different properties in terms of maneuvering their ability to move, or fold, in the cell where motions are greatly restricted to small spaces.

## 7. Conclusions

From a large amount of work by many excellent scientists during the last 50 years, it is clear that macromolecular crowding, as found in cells, will affect protein properties and their reactions. We have recently made a new contribution to this field by discovering that macromolecular crowding affects protein *native states*, *in vitro* in terms of secondary structure content, size and shape. For spherical proteins with inherent plasticity in their native states, protein-crowder interactions may modulate local conformations at active sites. Surprisingly, for the football-shaped *Borrelia* protein VlsE, when macromolecular crowding effects couple with thermal/chemical denaturation, dramatic shape changes are observed by a combined *in vitro* and *in silico* studies. As a result, a collapsed spherical form of VlsE is populated and a hidden antigen becomes surface exposed. Taken together, it appears that the geometry of proteins is related to its folding and conformational behaviors, which may be amplified under cell-like conditions. This is also evident from our finding of an improvement of the correlation between folding speed and contact order with consideration of asphericity of a protein for a large set of unrelated proteins. Our work started with monodisperse crowding agents to mimic effects of macromolecular crowding. This is essential to give a first understanding of protein dynamics in heterogenous cellular milieus in a controlled manner. Others have however shown the importance of using mixtures of different crowding agents for optimization of biochemical reactions [56,57]. We plan to expand our investigations toward polydisperse solution conditions in the near future.

## Appendix

In this appendix, we provide some of the details regarding our coarse-grained models and the choice of energy functions for apoflavodoxin and VlsE, as well as the simulation techniques used in our studies.

### a) Coarse-grained models

A coarse-grained off-lattice C_α_ side-chain model (SCM) [42,58] is built on all-atomistic protein models from the protein data bank. Each amino acid (except glycine) is represented by two beads: a C_α_ bead from protein backbones and a side-chain bead positioned at the center of mass of each side chain. Ficoll^®^ 70 is modeled as a hard-core sphere while Dextran 70 is modeled as a sphero-cylindrical object, constructed by two Ficoll^®^ beads linked by a harmonic bond.

The potential energy of the system with proteins and crowders is *E**_p_* + *E**_pc_* + *E**_cc_* where *E**_p_* is the protein energy function, *E**_pc_* are interactions between protein and crowders and *E**_cc_* are interactions between crowders.

The potential energy of a protein is *E**_p_*=*E**_s_*+*E**_nonbonded_*. The structural energy (*E**_s_*) consists of bond-length potential (*E**_bond_*), bond-angle potential (*E**_angle_*), dihedral potential (*E**_dihedral_*), and chiral potential (*E**_c_*) that assigns the L-isoform handedness to amino acids.

(1) $$E_{\text{bond}}=\sum_{\text{bonds}}k_{b}{\left(r-r_{o}\right)}^{2}$$

(2) $$E_{\text{angle}}=\sum_{\text{angles}}k_{\theta }{\left(\theta -\theta_{o}\right)}^{2}$$

(3) $$E_{\text{dihedral}}=\sum_{\text{dihedrals}}^{C_{a}-C_{a}-C_{a}-C_{a}}k_{\varphi }^{\left(n\right)}[1-\text{cos}(n\cdot (\varphi -\varphi_{o}))]$$

(4) $$E_{c}=\sum_{\text{chiral}}\cdot \;k_{c}\cdot {\left(c-c_{o}\right)}^{2}$$

In the above, *φ* is the dihedral angle, *r* is the distance between two adjacent beads, and *θ* is the angle of three consecutive beads. *c* is the triple scalar product defined by $c=\vec{r}\;C_{\alpha }^{i}C_{sc}^{i}\cdot \left(\vec{r}\;C_{\alpha }^{i}C_{\alpha }^{i-1}\times \vec{r}\;C_{\alpha }^{i}C_{\alpha }^{i+1}\right)$. $C_{\alpha }^{i}$ and $C_{sc}^{i}$ are the C_α_ bead and the side chain bead of the *i*th residue, respectively, *k**_b_* = 100*ε*,*k**_θ_* = 20*ε*,*k**_φ_*^(1)^ = *ε*,*k**_θ_*^(3)^ = 0.5*ε*, *k*_c_ = 20*ε* and *ε*=0.6 kcal/mol. The equilibrium values of *φ**_ο_*,*θ**_ο_*,*r**_ο_* and *c**_ο_* are calculated from the crystal structures of the proteins from the protein data bank (www.rcsb.org). The PDB ID for VlsE is 1L8W and for apoflavodoxin is 2FX2.

The non-bonded interactions *E**_nonbonded_* include sidechain – backbone interactions (*E**_sb_*), backbone – backbone interactions (*E**_bb_**)* and sidechain – sidechain interactions (*E**_ss_*). For *E**_bb_* we use an angular-dependent energy function that captures directional properties of backbone hydrogen bonds:

(5) $$E_{bb}^{ij}=A(\rho ){E^{\prime }}_{bb}^{ij}$$

and

(6) $$A(\rho )=\frac{1}{{\left[1+B(1-{\text{cos}}^{2}\rho )\left(1-\frac{\text{cos}\rho }{\text{cos}\rho_{\alpha }}\right)\right]}^{2}}$$

where:

(7) $${E^{\prime }}_{bb}^{ij}=\varepsilon_{ij}\left[{\left(\frac{\sigma_{ij}}{r_{ij}}\right)}^{12}-2{\left(\frac{\sigma_{ij}}{r_{ij}}\right)}^{6}\right]$$

In eqn. (7), ε*_ij_* for backbone hydrogen bonding is 0.6 kcal/mol while σ*_ij_* is the hydrogen bond length equal to 4.6Å. *A(ρ)* measures the structural alignment of two interacting strands. *ρ* is the pseudo dihedral angle between two interacting strands of backbones and it is defined in ref [42]. *A(ρ)=1* if the alignment points to β-strands or α-helices. *ρ**_α_* is the pseudo dihedral angle of a canonical helical turn, 0.466 (rad). *B*=1.

For E_bs_ interactions between *i* and *j* a repulsive potential term is used:

(8) $$E^{ij}=\varepsilon {\left(\frac{\sigma_{ij}}{r_{ij}}\right)}^{12}$$

where σ*_ij_* = σ*_i_* + σ*_j_*, σ*_i_* and σ*_j_* are van der Waals radii of interacting beads. ε=0.6 kcal/mol. Equation 8 is used for *E**_pc_* and *E**_cc_* as well.

### b) Energy functions for coarse-grained side chain interactions

The choice of the energy function for *E**_ss_* depends on the protein under study. *E**_ss_* adopts the same form in terms of Lennard-Jones potential as eqn. 7. Solvent-mediated interaction, ε*_ij_*, carries the characteristics of a protein that should match with its behavior in experiments. σ*_ij_* *= f(*σ*_i_* *+* σ*_j_**)* where σ*_i_* and σ*_j_* are van der Waals radii and *f = 0.9* in order to avoid volume clashes in the side-chains.

If a protein that folds in a relatively two-state fashion with few intermediates, a modified Go-like energy function is used for protein folding studies [46]. It assigns attractive interaction to contacts found in the crystal structure otherwise repulsion is used to produce a funnel-like folding energy landscape. We assigned a Go-like energy function to the apoflavodoxin protein model.

However, for a VlsE protein that renders rich structural characteristics, a more sophisticated energy function that depends on sequence information should be used for ensemble studies. We implement a so-called Betancourt-Thirumalai “statistical potential” [59] in which the amplitude of the solvent-mediated interactions, ε*_ij,_* is dependent on the amino acid types.

### c) Simulation techniques

The thermodynamic properties of our coarse-grained systems are studied by molecular simulations using Langevin equations of motion. An in-house version of AMBER6 [60] is used, where the Langevin equations of motion are integrated in low friction limit. The Replica Exchange Method [61] is implemented to enhance the sampling efficiency by incorporating simulations in different temperatures. Thermodynamic properties are calculated with the use of the weighted histogram analysis method[62,63]. Simulation details regarding time steps can be found elsewhere [13].
